# The Radiation Sterilization of Ertapenem Sodium in the Solid State

**DOI:** 10.3390/molecules24162944

**Published:** 2019-08-14

**Authors:** Karolina Kilińska, Judyta Cielecka-Piontek, Robert Skibiński, Daria Szymanowska, Andrzej Miklaszewski, Kornelia Lewandowska, Waldemar Bednarski, Mikołaj Mizera, Ewa Tykarska, Przemysław Zalewski

**Affiliations:** 1 Department of Pharmacognosy, Poznan University of Medical Sciences, Święcickiego 4, 60-781 Poznań, Poland; 2 Department of Medicinal Chemistry, Medical University of Lublin, Jaczewskiego 4, 20-090 Lublin, Poland; 3 Department of Biotechnology and Food Microbiology, Poznan University of Life Sciences, Wojska Polskiego 48, 60-627 Poznań, Poland; 4 Institute of Material Science and Engineering, Poznan University of Technology, Jana Pawła II 24, 60-965 Poznań, Poland; 5 Institute of Molecular Physics, Polish Academy of Sciences, Smoluchowskiego 17, 60-179 Poznań, Poland; 6 Department of Chemical Technology of Drugs, Poznan University of Medical Sciences, Grunwaldzka 6, 60-780 Poznan, Poland

**Keywords:** ertapenem, radiation sterilization, Q-TOF, microbiological activity, FT-IR, HPLC, EPR, XRPD, Raman

## Abstract

For the first time, the influence of ionising radiation on the physicochemical properties of ertapenem in solid state was studied. During our studies, we evaluated the possibility of applying radiosterilization to obtain sterile ertapenem. Spectroscopic (Fourier Transform Infrared (FT-IR)), thermal (differential scanning calorimetry (DSC), chromatography (High-Performance Liquid Chromatography (HPLC) and HPLC-MS), and X-ray powder diffraction (XRPD) studies shown that irradiation of ertapenem with the 25 kGy, the dose required to achieve sterility, does not change the physicochemical properties of the studied compound. The antimicrobial activity of ertapenem irradiated with the dose of 25 kGy was only reduced for one species. Based on the received results, we can conclude that radiostelization is a promising alternative method of obtaining sterile ertapenem. In our studies, ertapenem was also exposed to e-beam radiation with a dose of 400 kGy. It was determined that two novel degradation products that are structurally differently to degradants formed during hydrolysis and thermolysis.

## 1. Introduction

Ertapenem in the form of the sodium salt (ERP) is an analogue of the carbapenem—a β-lactam antibiotic. ERP has a broad spectrum of antibacterial activity against many Gram-positive and Gram-negative aerobic and anaerobic microorganisms, however it has restricted activity against *Acinetobacter* species, *Pseudomonas aeruginosa*, methicillin-resistant staphylococci, and enterococci—nosocomial pathogens [1,2]. It was licensed for parenteral therapy for once daily use in November 2001 in the USA and six months later in Europe, and since then it has been successfully used in the treatment of community acquired pneumonia and complicated infections of skin and skin structure, urinary tract, abdominal, and pelvic cavity [1,2]. The mechanism of action is based on binding to PBPs (penicillin binding proteins) on the bacterial cell wall, thus inhibiting the synthesis of peptidoglycan, which is an essential component, the lack of which leads to the weakening and lysis of the cell wall and, consequently, the entire bacterial cell. ERP has favorable structural features, contains a critical 1β-methyl substituent, thus exhibiting stability to the renal enzyme—dehydropeptidase-1—and it does not require the addition of DHP-1 inhibitors in the clinical use. Ertapenem is stable against hydrolysis by a variety of β-lactamases, except metallo-β-lactamases [3,4]. The chemical structure of carbapenems makes them very susceptible to degradation under the condition of high temperature and humidity, which could lead to destruction of their main element, the β-lactam ring, which is responsible for the antibacterial activity [5,6]. For this reason, it is particularly important to choose the suitable sterilization method for ERP and irradiation seems to be the most advantageous approach. However, the use of this method requires detailed studies and confirmation of the impact of irradiation on the pharmaceutical properties and structure of the drug [7,8,9,10,11,12,13].

In this paper, the effect of ionizing radiation on ertapenem sodium in the solid state has been investigated. A standard recommended dose of irradiation (25 kGy) [14] and higher radiation doses (50–400 kGy) have been applied for understanding the process of potential changes in ERP after sterilization.

## 2. Results

Our study on radiostability of ertapenem was divided into two areas. The first was identification of ertapenem before and after irradiation based on analytical and biological methods. The second one aimed at evaluating the structure of the degradation products.

The identity evaluation for ERP was confirmed by means of spectroscopic (Fourier Transform Infrared (FT-IR)), thermal-differential scanning calorimetry (DCS) and X-ray powder diffraction (XRPD) studies.

**Figure 1:**
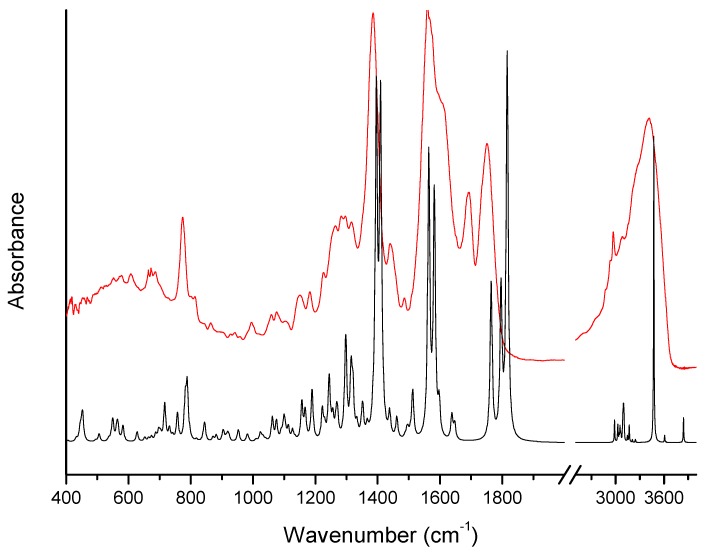
Calculated (black-DFT) and experimental (red) IR absorption spectra of ertapenem at room temperature.

**Figure 2:**
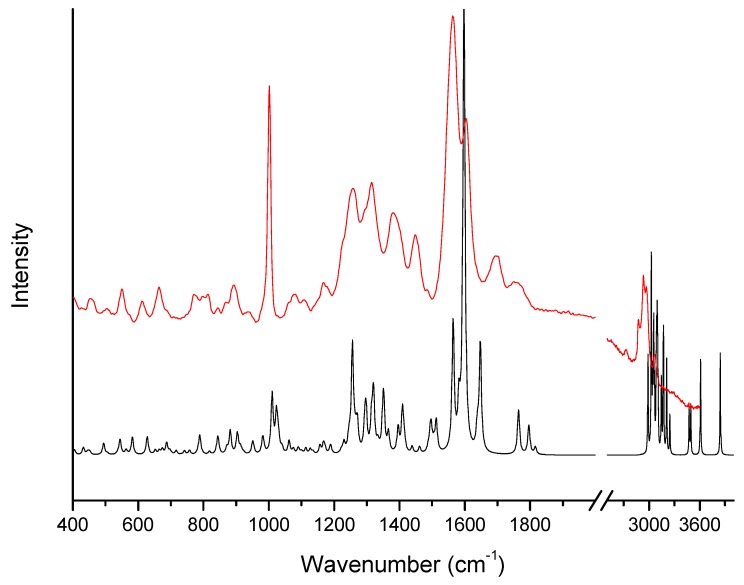
Calculated (black-DFT) and experimental (red) Raman scattering spectra of ertapenem at room temperature.

Figure 1 and Figure 2 show theoretical and experimental IR absorption and Raman scattering spectra of ertapenem, respectively. Theoretical calculation based on the density functional theory (DFT) approach was used in order to conduct particular spectroscopic analysis in regards to all characteristic bands.

The bands related to the respective groups/rings of ERP molecule can be distinguished in both the Raman scattering and IR absorption spectra. Accordingly, for the β-lactam ring, the bands are characteristically located in IR absorption spectrum at 675, 992, 1075, 1100, 1151, 1180, 1291, 1385, 1750, and 3263 and 3406 cm^−1^. The bands related to the vibration of the bonds in the β-lactam ring are also visible in Raman scattering spectrum and they are observed at 549, 816, 1077, 1100, 1169, 1382, 1601, and 1755 cm^−1^. The bands that are located in Raman spectrum at 549 and 816 cm^−1^ are associated with the deformation of the β-lactam ring. While the bands located at 1075 and 1077 cm^−1^ in IR absorption and Raman scattering spectra, respectively, are related to the breathing of the β-lactam ring. The bands at 1100, 1151, and 1169 cm^−1^ are mainly corresponding to the stretching vibration of the C-C bonds and C-O bonds in β-lactam ring and near the β-lactam ring in the COH and COOH groups. These bands are also additional components related to the wagging vibration of the C-H bonds (see Table 1).

The bands located at 1385 and 1382 cm^−1^ in IR and Raman scattering spectra, respectively, are related to the bending vibration of the C-O-H bonds in COOH group. Whereas, the band that was observed at 1613 cm^−1^ in IR absorption spectrum is corresponding to the stretching vibration of the C=C bond in β-lactam ring. At 1750, 1755 cm^−1^ are located the bands related to the stretching vibration of the C=O bond in COOH group. Moreover the bands located at 675, 992, 1180, and 1291 cm^−1^ are related to the deformation vibration out of plane of C-H bond in COOH group, stretching vibration of the C-S bond, wagging vibration of the C-H bonds, and stretching vibration of the C-N bond in the β-lactam ring. The bands mainly related to the stretching vibration of the C-C bonds and rocking, twisting, and stretching vibration of the C-H bonds are also visible for the pyrrolidine ring. Additionally, the bands located in Raman scattering spectrum at 940, 1258, 2883, and 2930 cm^−1^ are associated with the rocking twisting and stretching vibration of the C-H bonds. Whereas, the bands at 775, 892 cm^−1^ are related to the out of plane vibration of the N-H bond and stretching vibration of the C-C bond, respectively. The stretching vibration of the C=O bond in COONa group is located in the IR absorption spectrum in the massif between 1565–1690 cm^−1^ at about 1572 cm^−1^. In addition, the bands related to the stretching vibration of the C-N bond between pyrrolidine and benzene rings and bending vibration of the C-N-H bonds are also observed. They are located at 1057, 1315/1316, and 1565/1563 cm^−1^ in IR/Raman spectra. Whereas, the bands at 1228 and 1692/1695 cm^−1^ (IR/Raman) are corresponding to the stretching vibration of the C-C and C=O bonds between the pyrrolidine and benzene rings, respectively. Additionally, the band at 1656 cm^−1^ that was observed in IR absorption spectrum is related to the stretching vibration of the C=C bonds in benzene ring. The very wide band with maxima at 3263 and 3406 cm^−1^ related to the stretching vibration of the N-H and O-H bonds is also observed in IR absorption spectrum above 3000 cm^−1^.

**Figure 3:**
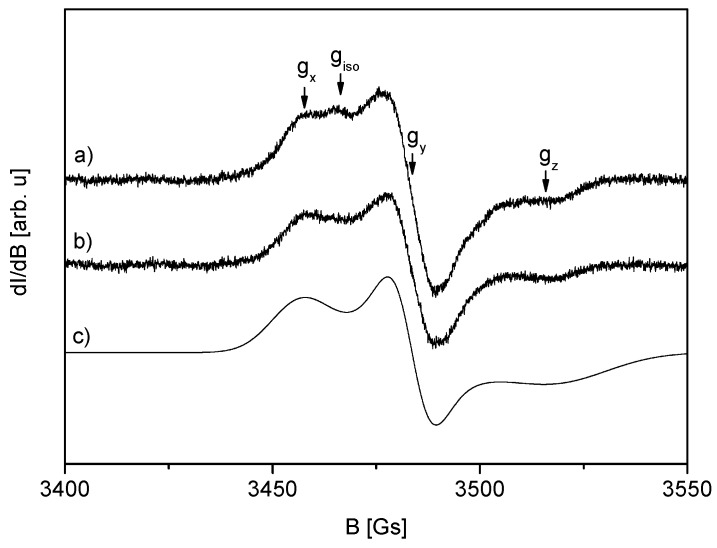
Electron Paramagnetic Resonance (EPR) spectra of the ERP sample recorded at 48.5 (**a**) and 355.5 (**b**) hours after irradiation (25 kGy) (**c**) presents the simulated anisotropic spectrum according to the parameters described in the text.

EPR studies indicate that there are no radicals in the non-irradiated material or free radical concentration is below the sensitivity of the spectrometer (<0.1 ppm). EPR spectrum of irradiated ERP (dose 25 kGy) consists of two different signals, as presented in Figure 3a. The first signal belongs to the free radical that is described by the following anisotropic values of spectroscopic coefficients: g_x_ = 2.021 (±0.001), g_y_ = 2.0048 (±0.0005), and g_z_ = 1.984 (±0.001). This signal was simulated in Figure 3c with the assumption of line width (ΔB) anisotropy: ΔB_x_ = 14 Gs, ΔB_y_ = 9 Gs, ΔB_x_ = 25 Gs. The second, much weaker, isotropic signal (Figure 3a) is described by the spectroscopic coefficient g_iso_ = 2.018 (±0.001), and it disappears several hundred hours after irradiation.

**Figure 4:**
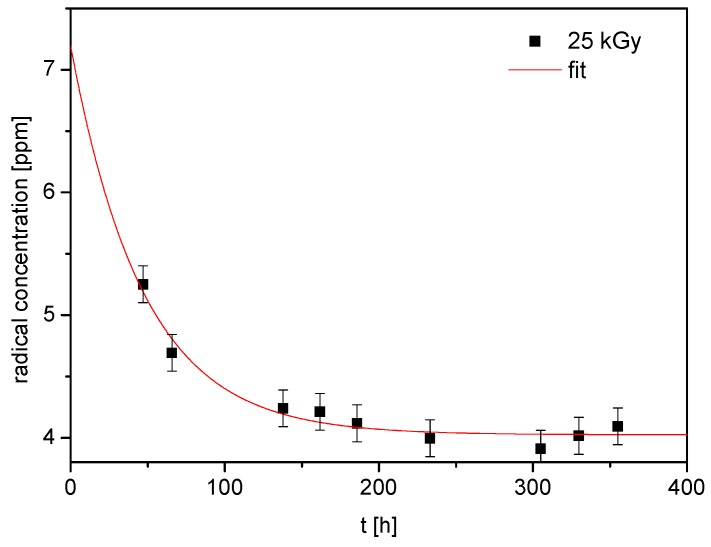
Concentration of free radicals vs. time after sterilisation (irradiation dose 25 kGy).

After irradiation, some of the radicals recombine (Figure 4) and their decay can be described by the following equation:

(1) $$I(t)=I_{s}+I_{n}e^{\frac{-t}{T}}$$

where *I(t)* is the total concentration of free radicals at any time *t* after irradiation, *I_s_* is the concentration of stable free radicals, *I_n_* is the concentration of unstable free radicals, and *T* is the mean lifetime of unstable radicals. After approximations of the equation (1) to the experimental points that are presented in Figure 4, the following parameters were obtained *I(t = 0h)* = 7.32 (±0.85) ppm, *I_s_* = 4.02 (±0.05) ppm, *I_n_* = 3.3 (±0.8) ppm, and T = 47 (±11) h. Summarizing, the 25 kGy irradiation dose causes the formation of radical defects in ERT at a very low level, which does not exceed several ppm.

**Figure 5:**
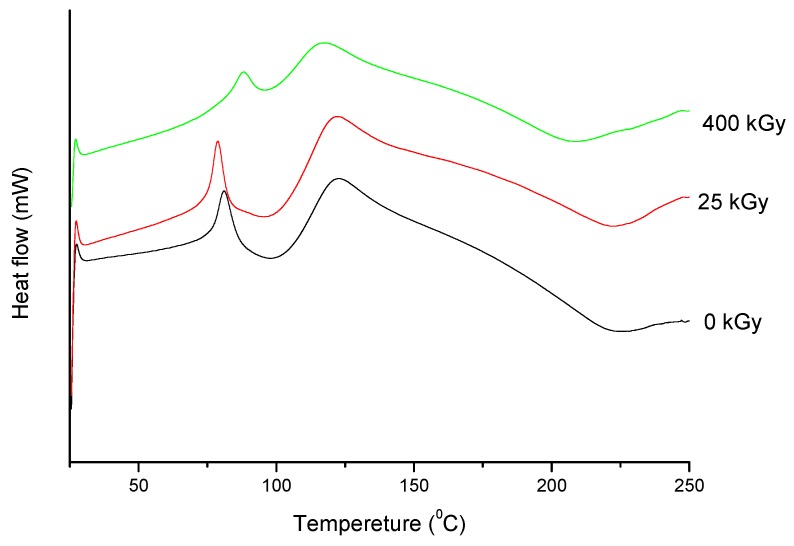
Differential scanning calorimetry (DSC) curves of non-irradiated and irradiated (25 and 400 kGy) ERP.

For the ERP sample irradiated at the dose of 25 kGy, the thermograms only show small changes in the peak position (ΔT_onset_ = 4.3 °C and ΔT_peak_ = 4.69 °C) in the comparison to the reference sample (0 kGy) (Table 2 and Figure 5). The results of melting enthalpies are also similar for both samples (ΔH = 52.1843 J/g for the non-irradiated sample and ΔH = 42.1795 J/g for irradiated at 25 kGy dose rate).

**Figure 6:**
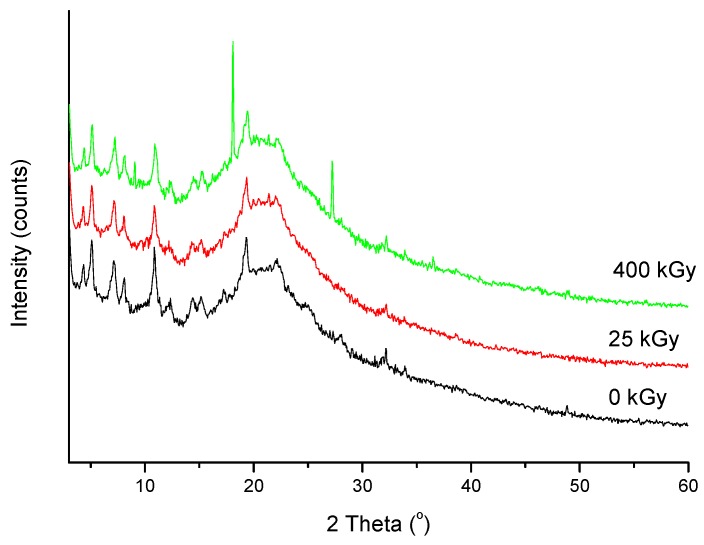
X-ray powder diffraction (XRPD) spectra of non-irradiated and irradiated ERT.

**Figure 7:**
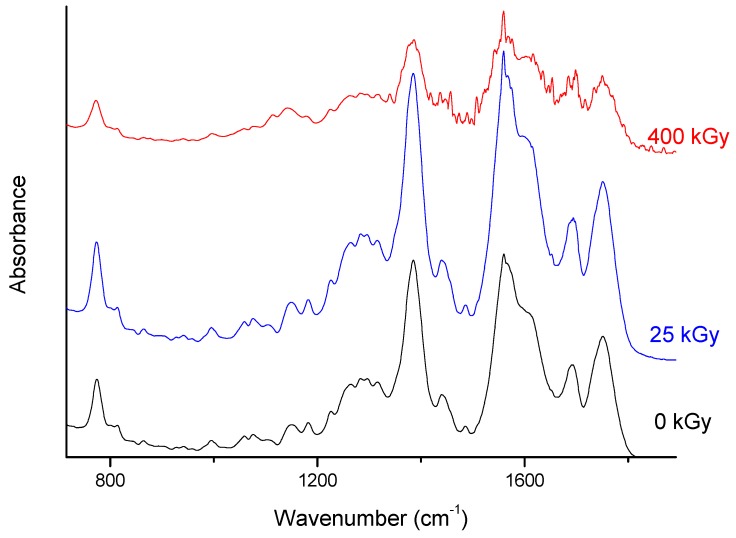
Fourier Transform Infrared (FT-IR) spectra of non-irradiated and irradiated ertapenem.

**Figure 8:**
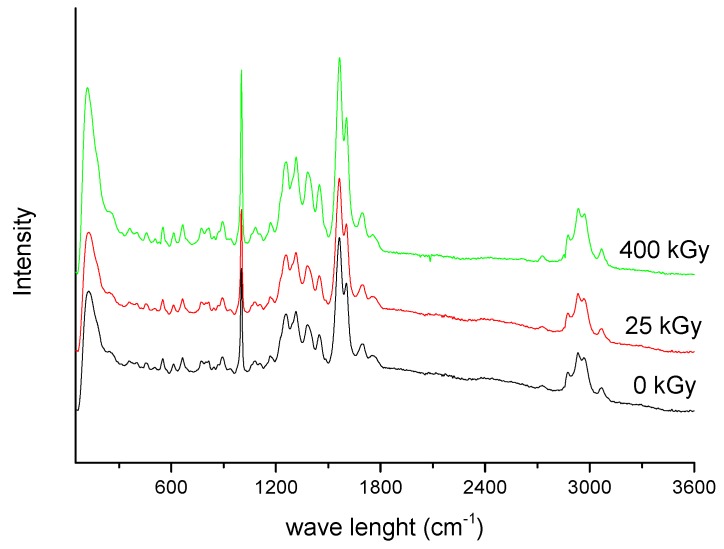
Raman spectra of non-irradiated and irradiated ERP.

However, for the 25 kGy irradiation dose there were no changes in High-Performance Liquid Chromatography-MS/MS (HPLC-MS/MS) or FT-IR, Raman, and XRPD spectra (Figure 6, Figure 7 and Figure 8).

In our study, a change in the color of samples after exposure to radiation—from white to yellow—was reported. In the next step, we verified the impact of primary packaging material on the ertapenem irradiation. None of the tested packages (PVP and glass containers) allowed for obtaining sterile ERP without color changes of the compound.

As shown in Figure 6, the XRPD diffractogram of the ERP sample that was irradiated with a dose of 400 kGy differs from the others (0 kGy, 25 kGy), which indicates the structural changes occurring in the material at higher doses of radiation. Additional diffraction peaks that were observed on the diffractogram of the ERP irradiated with a dose of 400 kGY indicate that there is a mixture of compounds in the sample.

At the next stage of the study, the minimum inhibitory concentration (MIC) of ERP against 10 strains of bacteria with pathogenic potential was analyzed. The analysis performed showed the highest MIC value for *Pseudomonas mirabilis*, *Escherichia coli*, *Salmonella typhimurium*, *Enterococcus faecalis*, *Salmonella enteriditis*, *Pediococcus pentosaceus*, *Staphylococcus aureus*, and *Pseudomonas aeruginosa* species (250 mg·L^−1^), while the lowest was determined for the *Acinetobacter baumanii* and *Enterobacter aerogenes* species (125 mg·L^−1^). ERP irradiation with the use of 25 kGy radiation only increased the MIC value for *Acinetobacter baumanii* (Table 3).

Beyond identifying the possible physical changes in the structure of irradiated ERP samples, its chemical stability was tested after exposition to 400 kGy. HPLC-diode array detector (HPLC-DAD) analysis of irradiated samples showed a loss of content of 11.5%. The RSD between the three measurements was 1%. The HPLC–MS/MS analysis showed two degradation products of ERP after its irradiation (400 kGy) (Table 4).

The molecular ions of ERP and its degradation products (E1 and E2) were very accurately identified (2.01, 4.56, and 1.63 ppm, respectively), which allowed for the calculation of the chemical formulas for the analyzed compounds (Table 4). The mass spectrometry/mass spectrometry (MS/MS) fragmentation spectra confirmed the proposed structures of the identified compounds (Table 4).

In addition, the bactericidal properties of ERP irradiated at 400 kGy were completely reduced for six bacterial strains (Table 3).

In the absorption spectrum for the ERP sample after irradiation with 400 kGy, more bands are observed in Figure 8. It looks that the previously recorded bands for samples without irradiation were splitting. For example, in the range where the band at 1692 cm^−1^ is observed for ERP, after irradiation there are visible two bands at 1700 and 1684 cm^−1^. A similar change occurs for the band with a maximum at 1565 cm^−1^. After irradiation, we can distinguish at least four bands at 1615, 1576, 1559, and 1541 cm^−1^. In the range between 1400–1500 cm^−1^, we can also observe more maxima of bands at 1507, 1488, 1456, 1437, and 1419 cm^−1^. These changes may result from the degradation of the ERP structure after irradiation. However, in the Raman scattering spectra of ERP, no changes can be seen after irradiation (Figure 8). Thus, perhaps the changes recorded for the infrared absorption spectra are the result of the inferior quality of the spectrum, resulting perhaps from the presence of water.

## 3. Discussion

Previous research concerning the radiostability of β-lactams antibiotics have confirmed their susceptibility to degradation [7,8,9,10,11,12,13]. Test results—both identification methods and stability tests—carried out for solid ERP reflect this statement.

According to the described results, after the irradiation of the sample with a dose of 25 kGy, no significant changes in the structure of the compound were identified—referring to spectroscopic (FT-IR, Raman), thermal (DCS), X-ray powder diffraction (XRPD), and mass spectrometry (HPLC-MS/MS) studies. On the other hand, a little decrease of absorption was observed in UV spectra and most likely, it is connected with minor degradation of ERP. This fact was additionally confirmed in EPR studies, where the formation of free radicals was identified after irradiation with 25kGy—the result was at a very low level, which does not exceed several ppm. In addition, stability was assessed by HPLC-DAD analysis [15], which showed a slight decrease in the content.

The use of a high irradiation dose (400 kGy) on ERP strongly intensified the effect of ERP degradation and allowed for the identification of degradation products by HPLC–MS/MS analysis (Table 4). The radiolysis products were therefore formed as a decomposition of the bond connecting substituent of β-lactam ring. This change decreases the bactericidal properties of ERP and it has been confirmed in the carried out tests.

The literature data confirm that discoloration or yellowing is the most common problem that is encountered after radiosterilisation of solid drugs [11]. What is particularly important, after dissolving the ERP irradiated samples in water for injections, we get clear and colorless solutions. Therefore, the authors suggest that the change in the color of ERP in the solid state after exposure to irradiation may be related to the presence of radiolyzed water that is trapped in the crystal structure of ERP.

The microbiological study demonstrated the stability of ERP biological activity after irradiation with a dose of 25 kGy. An increase in a MIC value was identified for only one species—*Acinetobacter baumanii*—but the increase was only to the standard MIC level that is presented by other species. Only the application of 400 kGy radiation dose shows a significant reduction and even the disappearance of bactericidal properties of ERP, which corresponds to earlier results of studies showing the decomposition of the compound (β-lactam ring opening) and formation of degradation products.

The conducted studies confirm the thesis put forward—ERP in solid state shows stability after irradiation with the dose of 25kGy and it maintains its pharmacological properties. The application of a very high radiation dose (400kGy) allowed for understanding physicochemical changes taking place in the molecule, identifying the degradation products, and linking them to changes in the bactericidal properties of ERP. It also confirms the need to conduct research for each molecule separately due to its individual response to radiation in comparison with compounds from the same group of drugs [9,10,11].

## 4. Materials and Methods

### 4.1. Standards and Reagents

Ertapenem sodium (ERT) was obtained from Glentham Life Sciences (Wiltshire, United Kingdom); it is a white to off-white, weakly crystalline, sterile salt with purity > 90.0%. ERP is soluble in water and 0.9% sodium chloride solution. All other chemicals and solvents were obtained from Merck KGaA (Darmstadt, Germany) and they were of analytical grade. High quality pure water was prepared while using the Millipore purification system (Millipore, Molsheim, France, model Exil SA 67120).

### 4.2. Irradiation

The samples of ERP were weighted into colorless glass vials in an amount of 5 mg and closed with plastic stoppers. All of the vials were exposed to β-irradiation in a linear electron accelerator LAE 13/9 (9.96 MeV electron beam and 6.2 μA current intensity) until doses of 25 and 400 kGy were absorbed.

### 4.3. Fourier Transform Infrared (FT-IR) Spectroscopy

The spectra were determined while using Fourier Transform Infrared (FT-IR) spectrometer, IR Affinity-1 Shimadzu. The samples were prepared by mixing ERP with potassium bromide in proportions of 1 mg of sample to 300 mg KBr. Pellets were formed under pressure of 15 ton/cm^2^ with a barrel of 13 mm in diameter. Absorption spectra were recorded with resolution of 2 cm^−1^ within a wavenumber range from 4000 to 400 cm^−1^ (30 scans per spectrum).

### 4.4. Electron Paramagnetic Resonance (EPR) Spectroscopy

The detection of the level of free radicals was carried out while using a Bruker ELEXSYS 500 spectrometer (Bruker, Billerica, MA, USA). The irradiated and non-irradiated powdered samples of ertapenem were tested in quartz capillaries at temperature 24 °C (X-band, 9.7 GHz). EPR spectra were recorded as a first derivative of the absorption signal. The spectra were accumulated 10 times due to the very small EPR signal, and the free radicals number was subsequently calculated using the method described elsewhere [16].

### 4.5. High-Performance Liquid Chromatography (HPLC-DAD) Analysis

The kinetic studies of ERP samples and separation of degradation products were conducted by the use of the Dionex Ultimate 3000 analytical system that consisted of a quaternary pump, an autosampler, a column oven, and a diode array detector (Dionex, Sunnyvale, CA, USA). The LiChrospher RP18, 5 µm particle size, 250 mm × 4 mm was used as a stationary phase and the composition of 25 mM phosphate buffer—methanol (85:15 *v*/*v*)—mobile phase. The flow rate was 1.2 mL·min^−1^. The 5.0 mg of each ERP samples were dissolved in 25.0 mL of water and the injection volume for HPLC analysis was 10 µL. Separation was performed at temperature 30 °C and the wavelength of the diode array detector (DAD) was set at 294 nm [15].

### 4.6. HPLC-MS/MS Analysis

The analysis was conducted with use of an Agilent Accurate-Mass Q-TOF LC/MS G6520B system with a DESI ion source and an Infinity 1290 ultra-high-pressure liquid chromatography system consisting of a G4220A binary pump, a G1330B FC/ALS thermostat, a G4226A autosampler, a G4212A DAD, and a G1316C TCC module (Agilent Technologies, Santa Clara, CA, USA). The control of the system, data acquisition, and qualitative analysis were conducted with the use of the MassHunter workstation software B.04.00. Separations were performed on Hibar RP-18e (2 µm particle size, 50 mm × 2.1 mm (Merck). The initial mobile phase composition was methanol—0.05% acetic acid (5:95) during 2 min. Then gradient elution was used, starting from mobile phase composition ratio (10:90) after 2 min. to 50:50 within 10 min. and the flow rate of the mobile phase was 0.3 mL·min^−1^. The Q-TOF detector was tuned in the positive (4 GHz) and the main parameters were optimized, as follows: drying gas 10 L/min., nebulizer pressure 40 psig, gas temp. 300 °C, capillary voltage 3500 V, skimmer voltage 65 V, fragmentor voltage 200 V, and octopole 1 radio frequency voltage 250 V. The data were acquired in the auto MS/MS mode with the mass range 50–950 *m*/*z* and the acquisition rate 1.2 spectra/s (for MS and MS/MS data). The collision energy was calculated from the formula 2V (slope)*(*m*/*z*)/100 + 6V (offset) and maximum two precursors per cycle were selected with an active exclusion mode after 1 spectrum for 0.2 min. The reference mass correction was used and values 121.0508 and 922.0097 *m*/*z* were selected as lock masses to ensure the accuracy of measurements.

### 4.7. X-ray Powder Diffraction (XRPD)

An X-ray powder diffraction (XRPD) pattern was obtained by means of a PANalitycal Empyrean system with CuKα_1_ radiation (1.54056 Å) at a voltage of 45 kV and a current of 40 mA. The ERP sample was scanned from 3° to 50° 2θ while using a step size of 0.017° and the scanning rate 15 s/step with the sample spinning.

### 4.8. Microbiological Study

The MIC (Minimal Inhibitory Concentration) values have been determined for each reference strains from the American Type Culture Collection and clinical isolates to verify the antimicrobial activity of ERP. MICs was assayed while using the serial dilutions method on the Mueller–Hinton liquid medium (Merck, Germany), which follows the standards of the Clinical and Laboratory Standards Institute (CLSI) [17]. In tests, microbial culture with a standardized optical density was used. The experiments for ERP samples were run in triplicate.

## 5. Conclusions

The results of the performed studies allow for concluding that ERP in the solid state is resistant to ionizing radiation in a standard sterilization dose of 25 kGy, and this method can safely be used for sterilization and decontamination of this compound. Irradiation with higher dose (400 kGy) leads to the following effects: loss in content of active substance by about 11.5%, radiolytic degradation of ERP with 2 product, lack of microbiological activity for six strains.

## Figures and Tables

**Table 1:** Selected characteristic vibrionic features of ertapenem in the form of the sodium salt (ERP); (s-stretching, b-bending, w-wagging, t-twisting, oop-out of plane).

IR (cm^−1^)	RAMAN (cm^−1^)	DFT (cm^−1^)	Bands Assignment
	549	544	Def. β-lactam ring
	613	627	Def. all molecule
	663	687	Def. benzene ring
675		717	O-H oop. in COOH
775	775	788	N-H oop. in pyrrolidine ring
	816	818	Def. β-lactam ring
	892	881	C-C s + C-H w in pyrrolidine ring
	940	953	C-H r in pyrrolidine and benzene rings + C-C s in pyrrolidine ring
992		981	C-S s
	1001	1010	Breathing benzene ring + C-C s in C-C s + C-N s in pyrrolidine ring
1057		1062	C-N s between pyrrolidine and benzene rings + def. pyrrolidine ring
1075	1077	1075	Breathing β-lactam ring
1100	1100	1110	C-C s near β-lactam ring + C-O s in COH + C-H w near β-lactam ring
1151		1156	C-O s in COH + C-C s near β-lactam ring + C-H w near β-lactam ring
	1169	1167	C-O s in COOH + C-H w
1180		1189	C-H w near β-lactam and benzene ring
1228		1222	C-C s between pyrrolidine and benzene rings + C-H t near pyrrolidine ring
1262	1258	1268	C-H t near pyrrolidine ring
1291		1315	C-N s in β-lactam ring + C-H w near β-lactam ring
1315	1316	1320	C-N s between pyrrolidine and benzene rings
1385	1382	1395	C-O-H b in COOH
1441	1445	1438	C-H b in benzene ring
1565	1563	1564	C-N-H b
1572		1582	C=O s in COONa
1613		1597	C=C s in β-lactam ring
1656		1647	C=C s in benzene ring
1692	1695	1764	C=O s between pyrrolidine and benzene rings + C-N-H b
1750	1755	1816	C=O s in COOH and near β-lactam ring
	2726	2987	C-H s in
	2883	3055	C-H s in pyrrolidine ring
	2930	3171	C-H s in pyrrolidine ring
2976		3208	C-H s in benzene ring
3263		3474	O-H in COOH group + N-H s in pyrrolidine ring
3406		3843	O-H s + N-H s

**Table 2:** Melting enthalpies and characteristic temperatures of ERP from differential scanning calorimetry (DSC) data.

(kGy)	T_onset_ (°C)	T_endset_ (°C)	ΔH (J/g)
0	66.5	73.9	52.2
25	70.8	78.7	42.2
400	72.9	83.5	29.5

**Table 3:** MIC values (mg·L^−1^) of non-irradiated and irradiated ertapenem samples.

Microorganism	0 kGy	25 kGy	400 kGy
*Pseudomonas mirabilis* ATCC 12453	250	250	-
*Escherichia coli* ATCC 25922	250	250	-
*Salmonella typhimurium* ATCC 14028	250	250	250
*Acinetobacter baumanii* ATCC 19606	125	250	250
*Enterococcus faecalis* ATTC 29212	250	250	-
*Salmonella enteriditis* ATCC 13076	250	250	250
*Enterobacter aerogenes* ATCC 13048	125	125	-
*Pseudomonas aeruginosa* ATCC 27853	250	250	-
*Pediococcus pentosaceus*	250	250	250
*Staphylococcus aureus* ATCC 25923	250	250	-

**Table 4:** Quadrupole time-of-flight (Q-TOF) accurate mass elemental composition and mass spectrometry/mass spectrometry (MS/MS) fragmentation of the analyzed substances.

Chemical Structure	Retention Time	Measured Mass (*m*/*z*)	Theoretical Mass (*m*/*z*)	Mass Error (ppm)	Molecular Formula [M + H^+^]	MS/MS Fragmentation Ions (*m*/*z*)	MS/MS Fragment Formula
Ertapenem 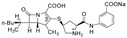	7.3	476.14955	476.14859	2.01	C_22_H_26_N_3_O_7_S	432.15889 346.12227 233.09189 181.07941 114.03702 68.05031	C_20_H_22_N_3_O_6_S C_17_H_21_N_3_O_3_S C_12_H_13_N_2_O_3_ C_9_H_11_NO_3_ C_6_H_10_O_2_ C_4_H_6_N
E1 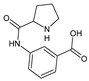	4.2	235.10871	235.10771	4.56	C_12_H_15_N_2_O_3_	99.98675 70.06612	C_5_H_12_N_2_ C_4_H_8_N
E2 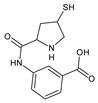	5.5	267.07880	267.07978	1.63	C_12_H_15_N_2_O_3_S	230.96416 182.03794 102.03734 68.04946	C_12_H_11_N_2_O_3_ C_8_H_10_N_2_OS C_4_H_8_NS C_4_H_6_N
